# Prediction of Antipsychotic Drug Doses for BPSD in Alzheimer’s Disease Using Deep Learning Techniques

**DOI:** 10.3390/diagnostics16121894

**Published:** 2026-06-18

**Authors:** Bo Hong, Tianli Tao, Yuhang Li, Zhen Gu, Han Zhang, Jianhua Chen, Ling Yue

**Affiliations:** 1 Shanghai Mental Health Center, Shanghai Jiao Tong University School of Medicine, Shanghai 200030, China; crystalhongbo@sjtu.edu.cn (B.H.); libai2000@sjtu.edu.cn (Y.L.);; 2 Alzheimer’s Disease and Related Disorders Center, Shanghai Jiao Tong University, Shanghai 200030, China; 3 Shanghai Institute of Traditional Chinese Medicine for Mental Health, Shanghai 201108, China; 4 Shanghai Clinical Research Center for Mental Health, Shanghai 201108, China; 5 Shanghai Key Laboratory of Mental Disorders Translational Research, Shanghai 201108, China; 6 School of Biomedical Engineering, ShanghaiTech University, Shanghai 201210, China; tianli.tao@kcl.ac.uk (T.T.); zhanghan2@shanghaitech.edu.cn (H.Z.); 7 Brain Health Institute at National Center for Mental Disorder, Shanghai 200030, China; 8 Department of Psychiatry, The First Affiliated Hospital of Xinjiang Medical University, Urumqi 830054, China; 9 Furong Laboratory, Xiangya Hospital, Central South University, Changsha 410078, China

**Keywords:** Alzheimer’s disease, BPSD, antipsychotic doses, structural MRI, deep learning, transfer learning, cascaded ResNet

## Abstract

**Background/Objectives**: Antipsychotic dosing for behavioral and psychological symptoms of dementia (BPSD) in Alzheimer’s disease remains empirical and variable. This study develops a deep learning model to predict individualized antipsychotic doses from structural MRI. **Methods**: A transfer learning approach with a cascaded ResNet (Cas-ResNet) was used. The model was first pre-trained on a large healthy aging dataset (CBMFM, *n* = 646) for brain age prediction, then fine-tuned on a BPSD dataset (SMHC, *n* = 86) to predict the defined daily dose (DDD) of antipsychotics. Model interpretability was performed using Grad CAM to identify predictive brain regions. **Results**: The proposed model achieved a mean absolute error of 0.19 and a Pearson correlation of 0.66 between predicted and actual doses, outperforming baseline 3DCNN, VGG, and DenseNet. Key contributing regions included the left inferior temporal gyrus, right parahippocampal gyrus, right putamen, left middle temporal gyrus, and left caudate. **Conclusions**: This proof-of-concept study demonstrates that deep learning can predict personalized antipsychotic doses from structural MRI, offering an objective tool to standardize BPSD pharmacotherapy and reduce empirical prescribing. The identified brain regions provide neurobiological insights into treatment response.

## 1. Introduction

The global demographic shift towards an older population has made Alzheimer’s disease (AD) one of the most significant and rapidly growing public health challenges [1]. Although cognitive decline defines AD, it is often complicated by Behavioral and Psychological Symptoms of Dementia (BPSD)—affecting up to 75% of patients. BPSD includes emotional dysregulation, psychosis, and aberrant motor behaviors [2,3]. These symptom clusters impose a heavy burden: patients suffer reduced quality of life, while caregivers face emotional, financial, and physical strain [4]. In clinical practice, when severe BPSD and safety risks exist, antipsychotic medication is needed for treatment [5]. However, most antipsychotics lack approval for BPSD or dementia subtypes [6], leading to widespread off-label use. Moreover, the FDA’s black-box warning cites a 60–70% increased risk of cerebrovascular events and all-cause mortality in this population [7].

Consequently, clinicians must undertake a complex risk-benefit assessment. The decision to prescribe requires weighing the urgent need to control dangerous or distressing symptoms against the significant risk of serious adverse events [8]. This challenge is exacerbated by the absence of standardized, evidence-based treatment protocols for dosing. Current practice relies on a ‘start low, go slow’ approach, which is inherently empirical and subject to wide inter-clinician variability. This trial-and-error method can lead to two undesirable outcomes: sub-therapeutic dosing, which fails to control symptoms and prolongs patient suffering, or accidental overdosing, which exposes patients to unnecessary risks, including excessive sedation, falls, and the life-threatening adverse events cited in the black-box warning. The development of an objective, individualized, and precise method for predicting antipsychotic dosage is therefore a critical unmet need to standardize medication and improve the safety of BPSD management.

Neuroimaging, especially structural MRI, provides a data-driven approach to this challenge. AD pathologies are linked to specific atrophy patterns [9]. Prior studies have associated BPSD with brain changes: apathy and psychosis relate to gray-matter loss or dysfunction in front-limbic circuits [10]; agitation and aggression correlate with frontal and subcortical limbic atrophy or hypoperfusion [11]. In terms of predicting drug efficacy or dosage, there are also some related studies on structural MRI. Brain age difference (BAG) can be used as a prediction of the efficacy of antipsychotic drugs in patients with schizophrenia (SZ) [12]. White matter (WM) density and functional connectivity (FC) of the right superior frontal gyrus (SFG) may predict the treatment outcome of antipsychotics for SZ [13]. However, there is currently no similar study on BPSD in AD patients.

Deep learning has enormous potential in deciphering complex patterns in imaging data. Deep convolutional neural networks (CNNs) have revolutionized neuroimaging analysis by learning complex, hierarchical features directly from raw scans. For instance, when classifying AD versus healthy controls on MRI, deep networks yield higher accuracy than traditional (hand-crafted feature) methods [14]. 3D DenseNet was trained on thousands of brain MRI scans to predict each subject’s chronological age [15], demonstrating that layered architectures can capture subtle age-related atrophy patterns. A key challenge in applying deep learning to medical imaging is the limited size of annotated datasets, especially for niche clinical cohorts like BPSD. Transfer learning (TL) offers a common solution: pretrain on a large dataset, then fine-tune on domain-specific data. Typically, CNNs are pretrained on ImageNet and adapted to medical images [14]. For instance, researchers may first train a CNN to distinguish AD from controls using ADNI data, then reuse those weights (“local transfer”) to classify finer cognitive states (e.g., stable vs. progressive mild cognitive impairment). Such local-TL approaches reliably improve performance—models converge faster and achieve higher accuracy on secondary tasks [16]. TL reduces overfitting and training time compared to training from scratch. Despite these advances, existing TL applications in neuroimaging remain limited to classification or segmentation outputs, predicting discrete diagnoses rather than continuous clinical parameters. However, deep learning models are often criticized as ‘black boxes,’ which limits their clinical acceptance. Explainable Artificial Intelligence (XAI) techniques address this issue by generating saliency maps that highlight the input regions most influential for a model’s prediction, enabling clinicians to verify whether the model’s decisions align with known neurobiology. Among existing XAI methods, Gradient-weighted Class Activation Mapping (Grad-CAM) [17] is one of the most widely adopted approaches for convolutional neural networks.

In this study, we propose a transfer-learning approach to predict individualized antipsychotic doses from structural MRI. First, we pre-train a Cascaded ResNet (Cas-ResNet) on a large healthy aging MRI dataset using brain age prediction as the source task, enabling the model to learn generalizable brain morphology features. We then fine-tune this model on our smaller BPSD dataset for drug dose prediction. This strategy effectively transfers knowledge from large to small data [18]. Additionally, we apply model interpretability to identify key brain regions, offering novel insights into BPSD neurobiology and personalized interventions.

## 2. Materials and Methods

### 2.1. Study Design and Transfer Learning Framework

**Figure 1:**
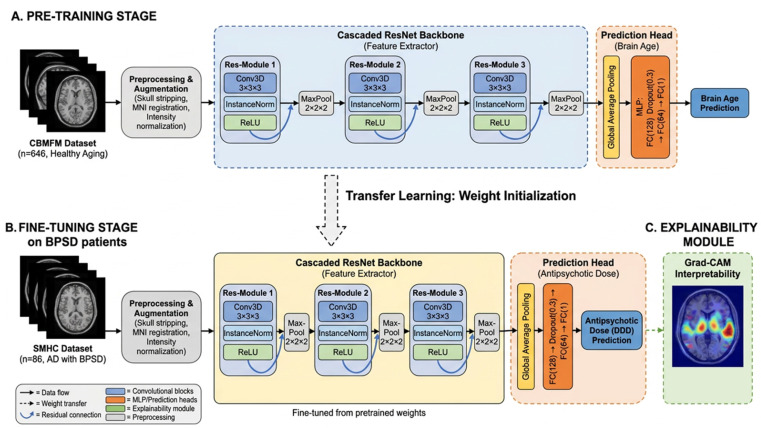
Schematic workflow and network architecture of our proposed method.

Our methodology employs a two-step transfer learning process, as shown in Figure 1. First, we pre-train our model on a large MRI dataset from a healthy aging population (CBMFM dataset). We chose brain age prediction as the pre-training task, as age is highly correlated with brain morphological changes, enabling the model to learn fundamental, generalizable features of brain structure. Subsequently, this pre-trained model is fine-tuned using our smaller, specialized BPSD dataset (SMHC dataset) to predict antipsychotic drug doses. This strategy leverages the robust feature representation learned from the large dataset to achieve high accuracy on the target task, even with limited clinical data.

### 2.2. Datasets

#### 2.2.1. CBMFM Dataset

The dataset used to pretrain the model was collected from the ongoing Chinese Brain Molecular and Functional Mapping (CBMFM) project, aiming to develop the brain templates of Asians in the aging process [19]. Specifically, we used 3D high-resolution T1-weighted MRI of 646 healthy subjects (334 females and 312 males, ages 18–82). None of them reported a history of psychological or neurological disorders. According to the approved Institutional Review Board protocol (No. 2019-105-01), all the volunteers signed informed consent forms for participating in this study. All the T1-weighted MRI data in CBMFM dataset were acquired using 3.0 T scanner (uMR790, United Imaging, Shanghai, China) at four sites with standardized acquisition parameters (TR = 2300 ms, TE = 2.98 ms, flip angle = 9°, voxel size = 1 × 1 × 1 mm^3^).

The CBMFM project is a longitudinal investigation of neurocognitive changes in over 3000 individuals between 20 and 80 years old with no neurological or psychiatric disorders. By pretraining our deep learning models on this large normative dataset spanning multiple decades of the adult lifespan, we could learn the patterns and trajectories of healthy structural brain aging. This provided a comprehensive baseline representation of the normal aging process against which to detect neurobiological deviations associated with neuropsychiatric conditions. Leveraging the CBMFM dataset allowed us to disentangle the neural signatures of BPSD pathology from the effects of normative brain aging. With a solid pretraining foundation modeling preserved brain integrity over the lifespan, we could then better isolate and characterize the specific neural alterations underlying BPSD psychopathology and medication dosage requirements in our patient cohort.

#### 2.2.2. SMHC Dataset

Our primary dataset for drug dose prediction was collected at the Shanghai Mental Health Center (SMHC). It included T1-weighted MRI scans from 86 AD patients with BPSD (28 males, 58 females). BPSD patients met the following inclusion criteria: (a) The diagnosis of AD according to the International Classification of Diseases, Tenth Revision (ICD-10) criteria. (b) Presence of clinically significant behavioral and psychological symptoms as assessed by the Neuropsychiatric Inventory (NPI) score ≥ 4. (c) Ability to tolerate MRI scanning. (d) There were no limitations with respect to age or sex. Exclusion criteria were as follows: (a) Delirium or acute stress reactions. (b) Depressive disorders and other psychiatric conditions, along with alcohol or drug dependency, poisoning, and genetic or metabolic anomalies. (c) Other neurodegenerative diseases leading to dementia. (d) Major systemic disorders (e.g., cardiovascular, respiratory, hepatic, renal, or hematopoietic disorders). (e) Past occurrences of organic brain diseases, brain injuries, or neurosurgery. (f) Contraindications to MRI (metallic implants, claustrophobia).

Their cognitive function was evaluated using the Mini-Mental State Scale (MMSE), and the behavioral and psychological symptoms were evaluated by the Neuropsychiatric Inventory (NPI). 3D T1w brain structural MRI data were acquired at baseline. The demographic and neuropsychological information of the participants is in Table 1. All patients underwent standardized clinical treatment (with or without concomitant antipsychotic drugs) and were followed up until BPSD remission. Drug treatment procedures (including drug types and doses) were recorded. To create a standardized label for our model, the antipsychotic drugs (risperidone, quetiapine, etc.) of each patient were converted using a defined daily dose (DDD) method [20] to obtain the final daily dose. This resulted in a continuous dose label ranging from 0.1 to 1.2, which served as the ground truth for model training and evaluation.

### 2.3. Data Preprocessing

A standardized preprocessing pipeline was applied to all T1-weighted images to ensure consistency and quality. The pipeline included: (1) skull stripping using FSL’s Brain Extraction Tool (BET) [21], (2) intensity normalization to correct for signal variations, and (3) spatial registration to the MNI152 standard space using the ANTs 2.5.0 toolkit. To maintain consistency across datasets, all images were resampled to 1 × 1 × 1 mm^3^ isotropic voxels and cropped to a uniform size of 182 × 218 × 182 voxels. A final manual quality control step was performed to exclude any images with significant motion artifacts or registration failures.

### 2.4. Network Architecture

Our model’s architecture was carefully designed to address the unique challenges of analyzing 3D neuroimaging data for a complex prediction task. The core of our design is a Cascaded Residual Network (Cas-ResNet), which synergistically combines the strengths of two powerful deep learning concepts: Residual Networks (ResNets) and cascaded architectures.

The foundation of our network is the Residual Network (ResNet) framework. A primary challenge in training deep neural networks is the vanishing gradient problem, where gradients diminish as they propagate back through many layers, hindering effective learning. ResNet addresses this by introducing “shortcut” or “skip” connections that allow gradients to bypass one or more layers. This innovation enables the training of substantially deeper networks, which are essential for capturing the intricate and subtle patterns present in high-resolution MRI data, without sacrificing training stability.

While ResNet provides the depth, a cascaded architecture provides a strategic, hierarchical approach to feature extraction. Instead of processing the entire image volume in a single, monolithic block, a cascaded design breaks the problem into a sequence of stages. The initial stage processes the input image to learn coarse, global features (e.g., overall brain shape and atrophy patterns). Subsequent stages then receive the feature maps from the previous stage and progressively refine the analysis, focusing on increasingly finer and more localized details (e.g., the specific morphology of the hippocampus or cortical thickness). This coarse-to-fine strategy is highly effective for anatomical data, as it allows the model to build a rich, multi-scale understanding of brain structure.

By integrating these two principles, our Cascaded ResNet (Cas-ResNet) leverages the best of both worlds. The cascaded structure organizes the learning process into a logical, multi-scale hierarchy, while the residual connections within each stage ensure that the network can achieve the necessary depth to extract meaningful features at each level of analysis. This combined architecture is not only powerful in its representational capacity but also parameter-efficient, making it particularly well-suited for our study, where robust feature learning must be achieved with a limited clinical dataset.

The Cas-ResNet architecture consists of three cascaded residual modules as a feature extractor and a final prediction module. Each residual module contains three sequential blocks comprising 3D convolution layers (kernel size 3 × 3 × 3, stride 1 × 1 × 1), instance normalization, and ReLU activation. The number of feature maps progressively increases from 64 to 128 to 256 across the three modules, with max pooling (2 × 2 × 2) applied after each module to reduce spatial dimensions.

The final prediction module includes two cascaded Multi-Layer Perceptron (MLP) layers with 128 and 64 neurons respectively, followed by a single output neuron for dose prediction. Dropout (*p* = 0.3) was applied between MLP layers to prevent overfitting.

For the age prediction task, we employed mean squared error (MSE) loss and AdamW optimizer with a learning rate of 0.001 and a batch size of 8. The learning rate was controlled by an exponential scheduler with decay factor γ = 0.5. The CBMFM dataset was randomly divided into training (80%) and validation (20%) sets. After pretraining, five-fold cross-validation was conducted on the SMHC dataset for drug dose prediction.

### 2.5. Feature Interpretability Analysis

To move beyond a “black-box” model and provide clinically relevant insights, we performed a group-level feature interpretability analysis using Gradient-weighted Class Activation Mapping (Grad-CAM). This technique generates saliency maps that highlight the brain regions most influential in the model’s dose prediction. Individual attention maps were registered to the MNI152 template using ANTs, smoothed with a 6 mm FWHM Gaussian kernel, and subjected to one-sample *t*-tests with age, gender, and education as covariates. Gaussian random field (GRF) theory was applied for multiple comparisons correction, with a stringent voxel-level threshold of *p* < 0.001 and a cluster-level threshold of *p* < 0.05.

### 2.6. Implementation Details

The implementation of our experiment was based on PyTorch 3.9. During the experiment, we conducted a 5-fold cross-validation, each run with 80% of the data for training and 20% for validation. The pretrained model was trained for 100 epochs. The parameters from the best epoch were selected as the initial weights for fine-tuning in the transfer learning. The transfer learning was conducted for 30 epochs, and the epoch with the best performance on the validation set was used to compute the results on the validation set. This is a regression task with a continuous output (DDD ∈ [0.1, 1.2]). Therefore, we used MAE and Pearson correlation between predicted drug doses and the calculated DDD (real drug dose) as evaluation metrics.

## 3. Results

### 3.1. Model Performance

We used 3D Convolutional Neural Networks (3DCNN), VGG, and DenseNet as the baseline algorithms to assess whether the Cas-ResNet model outperforms other baseline models. The result is summarized in Table 2. Our proposed approach already exhibited superior results compared to these models even without pretraining. These results collectively highlight the effectiveness of our model in capturing subtle symptom-related features and in projecting the features to drug dose. Note that our lightweight model had more accurate predictions with fewer parameters than similar models. This outcome might be attributed to the advantage of the lightweight model in small datasets. Traditional deep learning models, however, require more data for training, which is not feasible for many disease prediction tasks in clinical scenarios.

**Figure 2:**
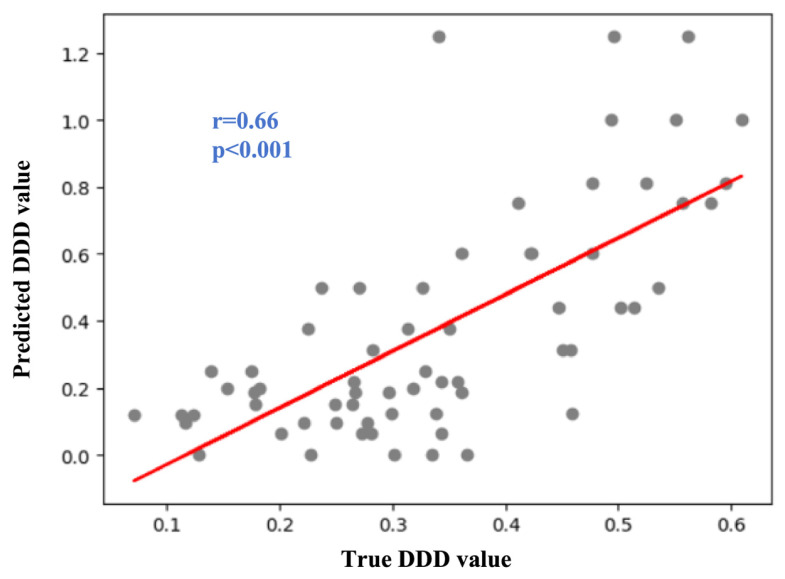
Scatterplot of drug dose prediction (*y*-axis) over the ground truth (*x*-axis). The red line represents the linear regression fit between the predicted and true DDD values.

Furthermore, we explored the performance of the pretrained version of our model. The results showed that the pretrained Cas-ResNet achieved better results with even fewer training epochs, with a very good Pearson correlation (0.66, see Figure 2 for the scatter plot). This could be attributed to the pretraining process that enables the model to capture substantial information from MRI. Moreover, the pretrained model requires fewer parameters and holds the potential for rapid deployment in future clinical settings with different prediction and diagnosis targets.

### 3.2. Feature Interpretation

**Figure 3:**
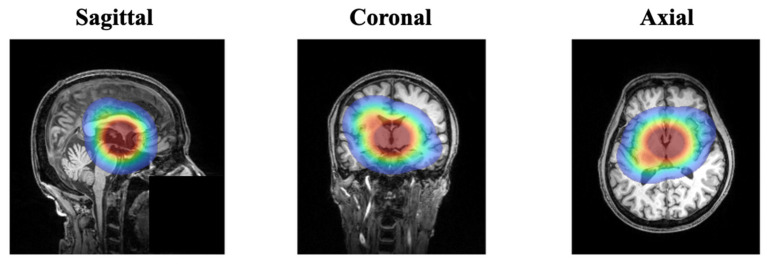
A showcase of individual-level Grad-CAM heatmap overlaid on the corresponding T1-weighted MRI. The color maps highlight the anatomical regions that most strongly drive the model′s BPSD drug dose predictions.

**Figure 4:**
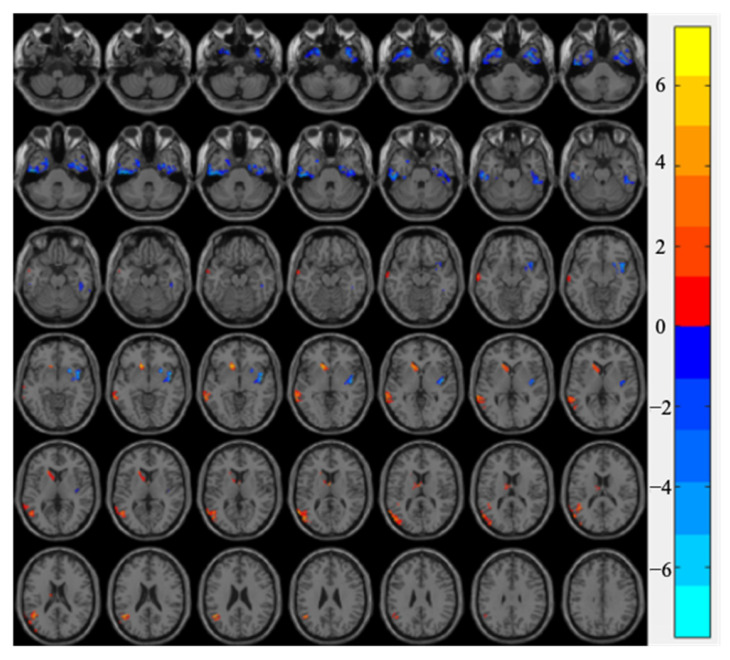
The significantly contributing brain regions identified by our method for BPSD drug dose prediction.

With feature interpretability analysis, we identified the brain regions related to the prediction of BPSD drug dose, providing valuable insights for precision medicine and the neuro-mechanism exploration for BPSD. Figure 3 shows the individual-level subject-smoothed Grad-CAM attention map. The figure clearly demonstrates that the model’s predictions are driven by anatomically meaningful regions, supporting the biological plausibility of our approach. Figure 4 shows five clusters that significantly contributed to drug dose prediction for BPSD. The color scale represents t-statistics derived from one-sample t-tests conducted on the group-level Grad-CAM saliency maps, with age, sex, and years of education entered as covariates of no interest. Multiple comparisons were corrected using Gaussian Random Field (GRF) theory. Regions exhibiting larger absolute t-values reflect greater consistency and statistical robustness of Grad-CAM activations across subjects, indicating that the model places greater reliance on these regions when predicting antipsychotic drug dose.

Table 3 presents the five clusters that contributed most to the deep learning model’s prediction of antipsychotic dose. All clusters survived a significance threshold of *p* < 0.001 (corrected). The cluster centers (MNI coordinates) were localized to the left inferior temporal gyrus (BA20), right parahippocampal gyrus (BA20), right putamen (BA13/putamen), left middle temporal gyrus (BA21), and left caudate nucleus (subcortical, no BA assignment). These regions encompass both temporal cortices involved in the neurobiology of BPSD and striatal structures that are primary targets of antipsychotic drugs.

## 4. Discussion

This study represents a pioneering effort to bridge the gap between advanced neuroimaging analytics and the pressing clinical challenge of antipsychotic dosing in BPSD. We successfully developed and validated a deep learning model capable of predicting individualized drug doses from structural MRI data with high accuracy (MAE = 0.19, r = 0.66). This achievement offers a tangible solution to a long-standing clinical problem.

### 4.1. Clinical Implications and Future Utility

Current prescribing practices are largely empirical, relying on a “start low, go slow” heuristic that, while cautious, is fraught with inter-clinician variability and can prolong patient suffering or inadvertently lead to over-medication. Our findings provide preliminary proof-of-concept evidence that MRI-based deep learning may contribute to future data-driven pharmacotherapy research in BPSD. By providing an objective, individualized dose recommendation derived from the patient’s unique neuroanatomy, such a tool could standardize the initial phase of treatment, establishing a more consistent and evidence-based starting point for clinicians.

A critical question for clinical translation is whether the achieved prediction error (MAE = 0.19 in DDD units, ranging from 0.1 to 1.2) is acceptable from a practitioner’s perspective. In absolute terms, a mean deviation of 0.19 DDD corresponds to approximately 15–20% of the full dose range. For an individual patient, this error margin could mean, for example, a predicted dose of 0.8 DDD versus an actual prescribed dose of 0.6 DDD. However, there is no established “gold standard” for individualized antipsychotic dosing in BPSD. In this context, our model provides a consistent, objective starting point that, even with an MAE of 0.19, may be non-inferior to the average clinician’s initial dose estimate—especially considering that inter-clinician variability often exceeds this margin. From a safety perspective, the model’s errors were not systematically biased toward overdosing; the scatter plot (Figure 2) shows no clustering of over-predictions at the high end of the dose range.

Furthermore, the intended clinical use is not to replace physician judgment but to assist it. A clinician seeing a predicted dose of 1.1 DDD for a frail patient could override it, while an unexpectedly low prediction might prompt a review of target symptoms. Thus, the current accuracy, while improvable, already offers a clinically useful decision support tool that reduces unexplained variability. Future prospective studies should directly compare model-assisted dosing against routine medicine in a randomized design to formally establish non-inferiority and safety margins.

### 4.2. Neurobiological Insights into BPSD and Treatment Response

Our interpretability analysis serves two purposes: it checks whether the model’s behavior is biologically plausible, and it offers hypotheses about which brain regions may be relevant to antipsychotic treatment response in BPSD. It is important to emphasize that these findings reflect statistical associations learned from the data, not direct causal mechanisms.

Among all input features derived from structural MRI, the five brain regions with the highest predictive importance were the left inferior temporal gyrus (Temporal_Inf_L), right parahippocampal gyrus (ParaHippocampal_R), right putamen (Putamen_R), left middle temporal gyrus (Temporal_Mid_L), and left caudate (Caudate_L). The five brain regions identified fall into two major neural systems: the temporal–parahippocampal network (left inferior temporal gyrus, left middle temporal gyrus, and right parahippocampal gyrus) and the striatal network (right putamen and left caudate). This bipartite pattern is consistent with the emerging understanding that BPSD arises from the combined effects of temporal lobe degeneration, which may contribute to reality monitoring deficits and misattribution phenomena, and striatal dopamine dysregulation, which modulates reward processing, behavioral control, and psychotic symptom expression [22,23,24]. Previous research using voxel-based morphometry demonstrated that temporal lobe gray matter volume—including both fusiform gyri and the left middle temporal gyrus—is significantly positively correlated with antipsychotic treatment response [25].

The striatal regions (putamen and caudate) are major targets of the mesolimbic and mesocortical dopamine pathways. The dorsal striatum receives dense dopaminergic projections and expresses high levels of dopamine D2 receptors, which are the primary molecular targets of most atypical antipsychotics [26]. A prospective clinical trial in antipsychotic-naive AD patients found that higher baseline gray matter volumes of both the left and right putamina and the left parahippocampal gyrus were positively associated with treatment response of psychotic symptoms after six weeks of risperidone treatment [24]. The caudate nucleus also emerged as a key region. As a D2-rich structure involved in motor control, it is both a therapeutic target for antipsychotics and a site where extrapyramidal symptoms (EPS) originate [27]. The model’s attention to the caudate might reflect a learned association between its structural integrity and the risk–benefit trade-off in dosing. However, an alternative interpretation is that clinicians already adjust doses downward when patients show signs of EPS risk, and the model simply captures that prescription pattern. Finally, the insular cortex—a hub for interoception and emotion—was also highlighted, which is consistent with its role in emotional and behavioral regulation [28,29].

Our study extends the existing literature in three important ways. First, while previous studies focused on predicting treatment response categories (responders vs. non-responders) or symptom improvement magnitudes, our model directly predicts the required DDD, providing a clinically actionable continuous output. Second, the use of deep learning enabled the model to learn nonlinear interactions among brain regions without prespecified anatomical hypotheses, yet the regions that emerged as top predictors aligned closely with those identified in hypothesis-driven studies [24], supporting the biological validity of our approach. Third, the feature importance ranking reveals that temporal and striatal structures are more predictive of required dosage than broader measures such as global atrophy or cognitive scores, suggesting that these regions capture domain-specific information about the neural substrate of BPSD and the pharmacological target engagement of antipsychotic drugs.

### 4.3. Limitations

Despite its promising results, this proof-of-concept study has several limitations that frame important directions for future research. The primary limitation is the relatively modest sample size of our BPSD cohort (*n* = 86). Although our transfer learning approach was specifically designed to mitigate the challenges of small datasets, the model’s generalizability and robustness should be confirmed through validation on larger, multi-site cohorts in the future. Such studies would be essential to ensure that the model performs reliably across diverse patient populations, clinical settings, and imaging protocols. In conjunction with acquiring larger datasets, future work could also explore advanced data augmentation techniques tailored for 3D neuroimaging. Methods such as generative adversarial networks (GANs) to create synthetic yet plausible brain scans could artificially expand the training set, potentially improving model robustness, provided that anatomical realism is strictly maintained. Since all BPSD patients were recruited from a single clinical center, the model may have learned cohort-specific clinical features. Therefore, the current findings should be interpreted as preliminary evidence of feasibility rather than definitive evidence of clinical generalizability. Future studies should validate the model in larger, independent, multi-center cohorts with harmonized imaging protocols and standardized treatment documentation.

Another important limitation concerns the use of DDD as a unified dosing label. While the DDD method enables cross-drug standardization and is widely adopted in pharmacoepidemiology, it does not fully capture pharmacodynamic differences between antipsychotics. For example, equivalent DDD values for risperidone and quetiapine may not correspond to comparable clinical efficacy or side-effect profiles, particularly in elderly patients, where small dose variations can substantially influence sedation, fall risk, and extrapyramidal symptoms. Future work should consider drug-specific modeling or stratified analyses by individual agents.

Furthermore, the scope of our predictive model was constrained by its reliance on a single data modality—structural MRI. While brain atrophy is a powerful biomarker, a more comprehensive understanding of treatment response could be achieved by integrating multi-modal data. Future research should prioritize prospective, longitudinal studies. Tracking patients over time would not only validate the initial dose prediction but also enable the development of dynamic models capable of guiding dose titration and maintenance, moving closer to a truly adaptive and personalized treatment strategy.

## 5. Conclusions

In this study, we developed and validated a novel deep learning model that predicts individualized antipsychotic drug doses for BPSD patients from structural MRI scans. Our transfer learning approach, using a lightweight Cas-ResNet architecture, achieved high performance (MAE = 0.19, r = 0.66) and provided neurobiological insights by identifying dose-related brain regions, including the temporal lobe, limbic system, and basal ganglia.

This work provides preliminary evidence that structural MRI-based deep learning may support the future development of data-driven decision-support tools for BPSD treatment, but prospective and external validation is required before clinical use. By offering a brain-based dose recommendation, our approach has the potential to standardize prescribing practices and reduce dangerous inter-clinician variability. This work serves as a proof-of-concept for a new paradigm in psychopharmacology, where treatment decisions are personalized based on individual neural signatures. Future work will focus on prospective validation and integration of multi-modal data to bring this powerful tool from the laboratory to the clinic, ultimately delivering safer and more effective medication to a vulnerable patient population.

## Figures and Tables

**Table 1:** Demographic and neuropsychological information of the SMHC dataset.

Characteristic	Value
Age (year)	74.7 ± 8.8
Gender (male, %)	28 (32.6%)
Years of education (year)	10.7 ± 3.8
MMSE	12.9 ± 6.9
NPI	31.7 ± 24.0

**Table 2:** Prediction performance and model complexity.

Method	Modality	r (95% CI)	MAE (95% CI)	Epochs	Parameters
Linear Regression	Clinical Info	0.24 (0.13–0.35)	0.27 (0.25–0.29)	/	/
XGBoost	Clinical Info	0.29 (0.18–0.40)	0.25 (0.23–0.27)	/	/
Random Forest	Clinical Info	0.34 (0.23–0.45)	0.23 (0.21–0.25)	/	/
3DCNN	MRI	0.31 (0.20–0.42)	0.24 (0.22–0.26)	100	33 M
VGG	MRI	0.23 (0.12–0.34)	0.31 (0.29–0.33)	100	7.8 M
DenseNet	MRI	0.38 (0.28–0.48)	0.28 (0.26–0.30)	100	19.5 M
Cas-ResNet w/o pretrain	MRI	0.45 (0.36–0.54)	0.21 (0.19–0.23)	100	8.4 M
Cas-ResNet w pretrain	MRI	0.66 (0.59–0.73)	0.19 (0.17–0.21)	30	4 M

**Table 3:** Statistics and location of contributing brain regions.

Cluster	*p*-Value	Cluster Center	BA	AAL
1	<0.001 **	−42, −20, −34	20	Temporl_Inf_L
2	<0.001 **	27, −4, −33	20	ParaHippocampal_R
3	<0.001 **	24, 11, −7	13	Putamen_R
4	<0.001 **	−64, −60, 12	21	Temporal_Mid_L
5	<0.001 **	−14, 4, 14	/	Caudate_L

** *p* < 0.001.

## Data Availability

The datasets presented in this article are not readily available because the data are part of an ongoing study. Requests to access the datasets should be directed to bellinthemoon@sjtu.edu.cn. No AI tools were used in the preparation of this manuscript.

## Abbreviations

The following abbreviations are used in this manuscript:

AD	Alzheimer’s Disease
BPSD	Behavioral and Psychological Symptoms of Dementia
TL	Transfer Learning
CNN	Convolutional Neural Network
DDD	Defined Daily Dose
CBMFM	Chinese Brain Molecular and Functional Mapping
